# Assessing the risk of intracranial aneurysm rupture using computational fluid dynamics: a pilot study

**DOI:** 10.3389/fneur.2023.1277278

**Published:** 2023-12-22

**Authors:** Yajun Zhu, Rong Zou, Xiaochuan Sun, Xingwei Lei, Jianping Xiang, Zongduo Guo, Hai Su

**Affiliations:** ^1^Department of Neurosurgery, 1st Affiliated Hospital of Chongqing Medical University, Chongqing, China; ^2^ArteryFlow Technology Co., Ltd., Hangzhou, China; ^3^Yongchuan Hospital of Chongqing Medical University, Chongqing, China

**Keywords:** intracranial aneurysms, rupture risk, hemodynamics, computational fluid dynamics, SAHsubarachnoid hemorrhage

## Abstract

**Objective:**

This study compared 2 representative cases with ruptured aneurysms to explore the role of hemodynamic and morphological parameters in evaluating the rupture risk of intracranial aneurysms (IAs).

**Methods:**

CTA and 3-dimensional rotational angiography (3DRA) of 3 IAs in 2 patients were retrospectively analyzed in this study. Hemodynamics and morphological parameters were compared between a ruptured IA and an unruptured IA in case1, and between before and after aneurysm rupture in case 2.

**Results:**

In case 1, the ruptured aneurysm had larger morphological parameters including size ratio (SR), aspect ratio (AR), aneurysm vessel angle (*θ*_F_), Aneurysm inclination angle (*θ*_A_), Undulation index (UI), Ellipticity index (EI), and Non-sphericity Index (NSI) than the unruptured aneurysm. And oscillatory shear index (OSI) is also larger. Higher rupture resemblance score (RRS) was shown in the ruptured aneurysm. In case 2, the aneurysm had one daughter sac after 2 years. Partial morphological and hemodynamic parameters including SR, AR, *θ*_F_, *θ*_A_, UI, EI, NSI, OSI, and relative residence time (RRT) increased, and normalized wall shear stress (NWSS) was significantly reduced. RRS increased during this period.

**Conclusion:**

SR and OSI may have predictive values for the risk of intracranial aneurysm rupture. It is possible that WSS Changes before and after IA rupture, yet the influence of high or low WSS on growth and rupture of IA remains unclear. RRS is promising to be used in the clinical assessment of the rupture risk of IAs and to guide the formulation of treatment plans.

## 1 Introduction

**Figure 1:**
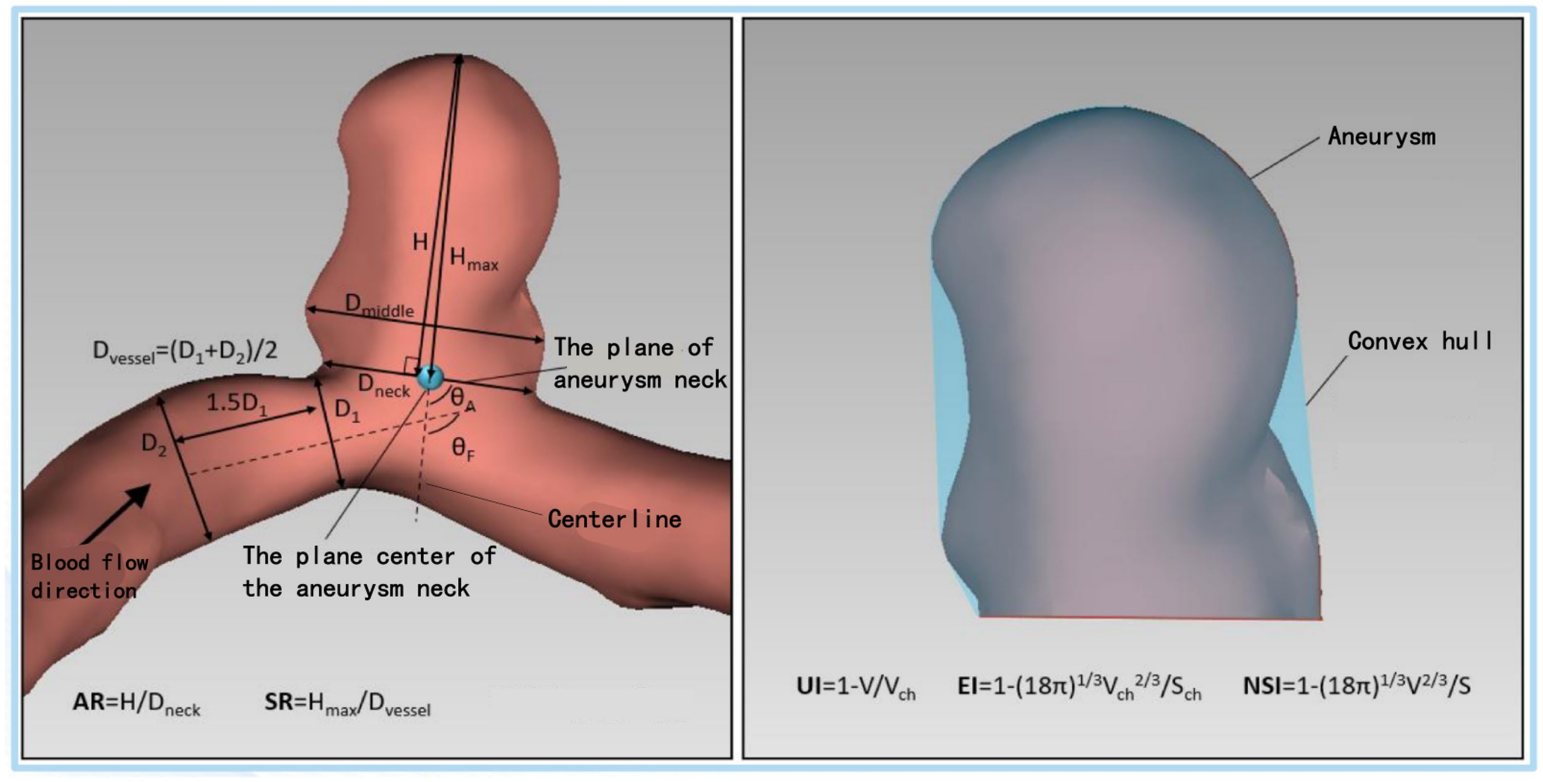
Schematic diagram of the morphological parameters of the aneurysm. *H*_max_, maximum aneurysm height; *H*, the vertical height of the aneurysm; *D*_middle_, maximum cross-section diameter parallel to aneurysm neck plane; *D*_neck_, diameter of the aneurysm neck plane; *D*_vessel_, the diameter of the vessel of the parent artery; SR, size ratio; AR, aspect ratio; *V*, aneurysm volume; *V*_ch_, the volume of the convex hull of an aneurysm; *S*, the surface area of the aneurysm sac; *S*_ch_, the surface area the convex hull; *θ*_F_, Aneurysm vessel angle; *θ*_A_, Aneurysm inclination angle; UI, Undulation index; EI, Ellipticity index; NSI, non-sphericity index.

Although the incidence of intracranial aneurysms (IAs) is less than 10% of the total population, ruptured IAs are characterized by high morbidity and mortality (1–3). With the development of imaging technology, the detection rate of unruptured IAs is getting higher, especially for small aneurysms, which increases pressure to diagnosis and treatment (4). On one hand, small IAs have a lower risk of rupture (5). But if they do rupture, it can cause significant harm and pose a risk to the patient’s life. On the other hand, surgical treatment for small IAs may result in potential complications, such as intraoperative and postoperative bleeding, vasospasm, cerebral infarction, and so on (6). Therefore, before formulating a treatment plan for an aneurysm, doctors must balance the rupture risk of the aneurysm and the risk of surgical complications (Figure 1).

At present, the treatment standard of IAs is still stratified according to the aneurysm size. For unruptured IAs, the clinical standard of surgical intervention is the aneurysm ≥7 mm in diameter (7). However, partial unruptured IAs less than 7 mm were found by imaging in clinic, and some of them still ruptured during the follow-up, causing a series of neurological damage and complications, even endangering the patient’s life (8, 9). In addition, although the risk of rupture of small IAs is currently low, our previous research (10) has shown that the proportion of ruptured small aneurysms is increasing every year. Therefore, the size of an aneurysm may be only one of the factors leading to its rupture, and the impact of hemodynamics cannot be ignored. When small aneurysms are detected, whether they will grow, change and rupture puzzles neurosurgeons and affects surgical decisions. Therefore, the size of aneurysm cannot completely guide clinicians to make surgical decisions on unruptured IA. Neurosurgeons need a more reliable evaluation method to guide the formulation of the treatment plan for unruptured small IAs.

The morphological and hemodynamic analysis of unruptured IAs provides great hope for the risk stratification of aneurysm rupture. The evaluation of the morphology and hemodynamics of IAs is mostly based on the establishment of the computational fluid dynamics (CFD) model of IAs, which aims to evaluate the morphological and hemodynamic factors of aneurysms that may cause growth, change and rupture of IAs (11, 12). In the past 10 years, CFD has been a potential research tool for studying many aspects of IAs, the most important of which is to analyze and study the growth and rupture mechanism of IAs and their relationship with vascular hemodynamics (12–16). Since this model is currently unable to fully simulate the actual situation of blood, it is still a long way from practical clinical applications.

Therefore, there are still many different arguments about clinical effectiveness and practicability of hemodynamics in assessing the risk of aneurysm rupture (17, 18). This study aims to verify the effectiveness of CFD by analyzing two ruptured aneurysms in follow-up observation.

## 2 Methods

### 2.1 Ethics approval and consent to participate

This study involving human participants were obtained consent from the patients and reviewed and approved by the Ethics Committee of the First Affiliated Hospital of Chongqing Medical University. The committee’s reference number: K2023-033.

### 2.2 Patient information

**Figure 2:**
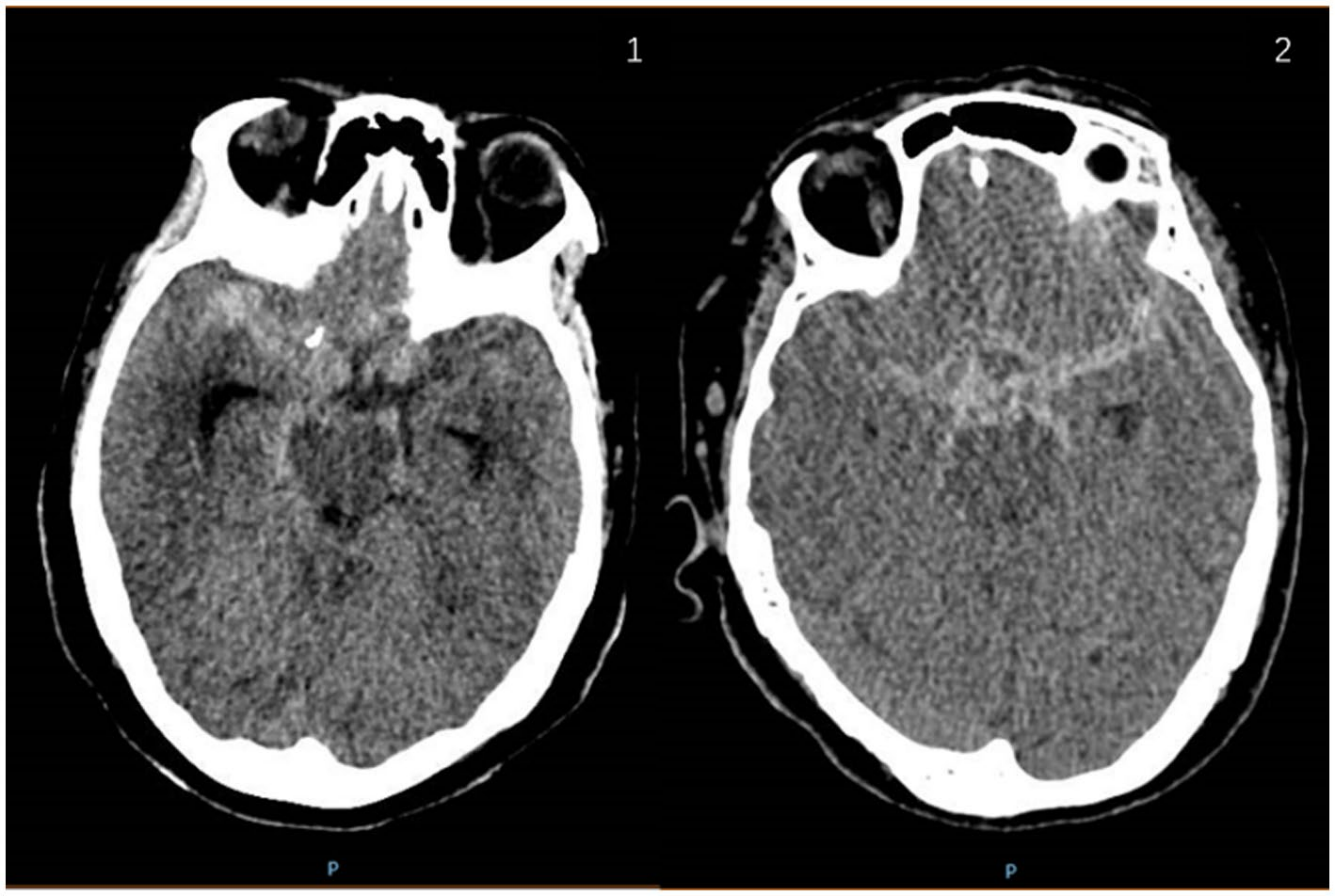
Head CT images of case 1 and case 2 with ruptured aneurysm and subarachnoid hemorrhage. 2.1: case 1; 2.2: case 2.

Case 1, an adult patient in 60 s, was admitted to the hospital due to bilateral posterior communicating artery aneurysm (PcoAA) found by head and neck CTA examination during physical examination. The left aneurysm was about 5.3 * 6.6 mm in size, and the right aneurysm was about 7.6 * 7.5 mm in size. It is planned to improve the head whole-brain angiography. During hospitalization, the aneurysm ruptured with a subarachnoid hemorrhage. According to the head CT (Figure 2) of the patient when the aneurysm ruptured, the right PcoAA was considered to be the responsible aneurysm.

Case 2, an adult patient in 70 s. Head and neck CTA examination revealed a left PcoAA in 2019, about 3.0 * 5.0 mm in size. The patient chose conservative treatment. In 2021, the patient was admitted to the hospital due to a sudden explosive headache, and the head CT (Figure 2) showed subarachnoid hemorrhage. According to the CT results, the left PcoAA was judged to be the responsible aneurysm, with a size of 5.0 * 5.3 mm.

### 2.3 Acquisition of morphological and hemodynamic parameters

The calculation of the morphological and hemodynamic parameters of each aneurysm’ 3DRA is described according to the description of Xiang et al. (19). Briefly, DICOM images were segmented in the region of interest, including aneurysm sac and adjacent parent vessels. Then eight morphological parameters (20, 21) were measured and calculated using AneuFlowTM (ArteryFlow, Hangzhou) (22), including *H*_max_, SR, AR, *θ*_F_, *θ*_A_, EI, NSI, UI. As for CFD models, finite volume meshes with 0.5–1 million elements were imported into the CFD solver to calculate time-resolved 3D velocity and pressure fields. Three pulsatile cycles were simulated, and the last cycle being taken as output to ensure that numerical stability. WSS and streamlines profiles were time-average over the third pulsatile cycle of flow simulation. The average values of NWSS of aneurysm sac, OSI, and RRT were calculated based on previous research (19). We scored the rupture risk of IAs, including RRSM, RRSH, and RRSC according to the RRS predictive regression model established by Xiang et al. (23).

## 3 Results: case-by-case description

**Figure 3:**
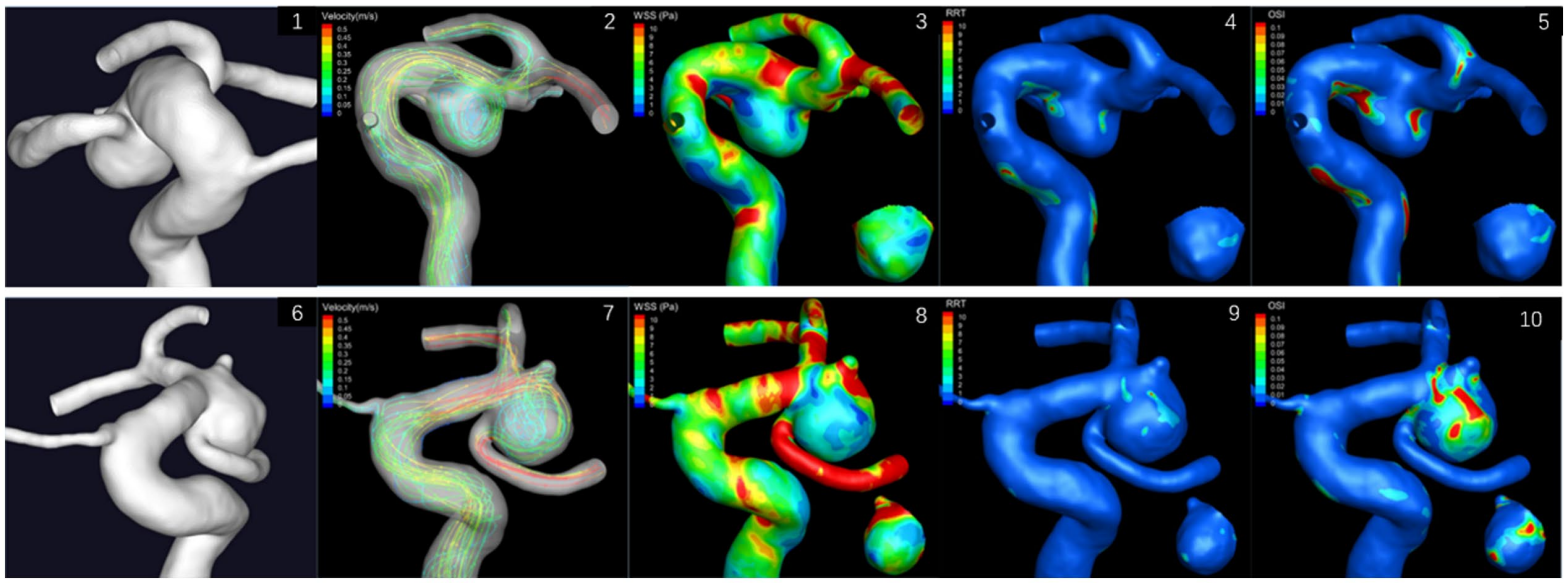
Hemodynamic results of bilateral posterior communicating aneurysms of case 1. The small image shows the aneurysm from the back view. 3.1–3.5: left aneurysm; 3.6–3.10: right aneurysm.

Morphological and hemodynamic analysis of the bilateral aneurysms of case 1 was conducted (Table 1). For morphological parameters, *H*_max_, *H*, *D*_middle_, *D*_neck_, volume, and surface area of the ruptured aneurysm were larger than those of the unruptured aneurysm, while the *D*_vessel_ was smaller. Therefore, the AR and SR of the ruptured aneurysm were larger than those of the unruptured aneurysm, and the SR of the ruptured aneurysm was 2.505, greater than the threshold value 1.75 reported previously (19). Both *θ*_F_ and *θ*_A_ of the ruptured aneurysm were larger, but not exceeding the threshold value (21). UI, NSI, and EI of the ruptured aneurysm were larger as well. In terms of hemodynamics, OSI of the ruptured aneurysm was larger, but NWSS and RRT of the unruptured aneurysm were larger (Figure 3). OSI had more significant difference. RRSM, RRSH, and RRSC of the ruptured aneurysm were all greater than 30%, while only the RRSH of the unruptured aneurysm was greater than 30%.

**Table 1:** Morphological and hemodynamics parameters of the aneurysms in case 1.

	Parameters	Position		Threshold
		Left	Right	
Morphology parameters of aneurysms	*H*_max_ (mm)	5.311	7.578	/
	*H* (mm)	5.138	6.865	/
	*D*_middle_ (mm)	6.649	9.143	/
	*D*_neck_ (mm)	6.589	7.446	/
	*D*_vessel_ (mm)	3.76	3.025	/
	*V* (mm^3^)	140.293	333.099	/
	*S* (mm^2^)	108.671	206.884	/
	*θ*_F_ (deg)	111.71	132.38	>118.25
	*θ*_A_ (deg)	82.4	89.36	>96.1
	AR (H/*D*_neck_)	0.78	0.922	>1.6
	SR (*H*_max_/*D*_vessel_)	1.413	2.505	>1.75
	UI	0.017	0.044	>0.09
	EI	0.044	0.097	>0.13
	NSI	0.046	0.108	>0.16
Hemodynamic parameters of aneurysms	NWSS	0.504	0.549	<0.39
	OSI	0.0073	0.0215	>0.0036
	RRT	0.405	0.387	>2.7
Rupture Resemblance Scores	RRS_M_	14.99%	40.55%	>30%
	RRS_H_	37.58%	86.77%	>30%
	RRS_C_	21.50%	81.20%	>30%

**Figure 4:**
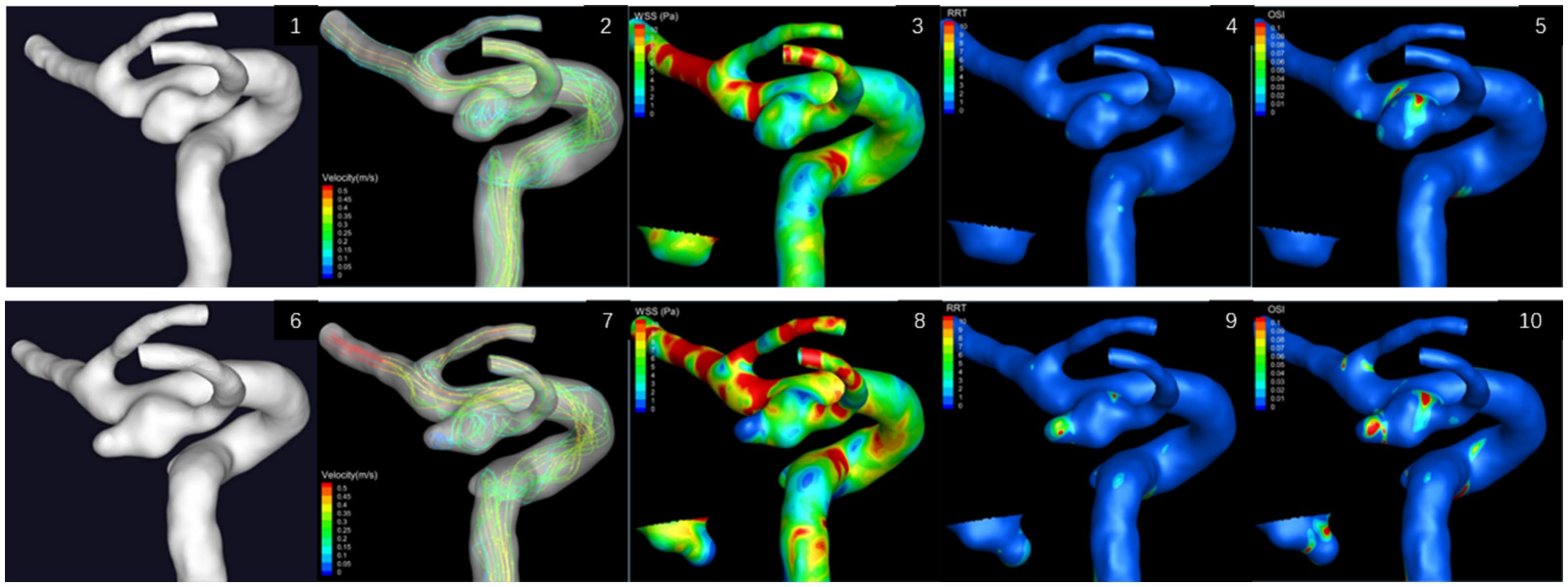
Hemodynamic results of left posterior communicating aneurysms in two stages of case 2. The small image shows the aneurysm from the back view. 4.1–4.5: in 2019; 4.6–4.10: in 2021.

In case 2, a daughter sac in the aneurysm was appeared in 2021 (Figure 4). Morphological and hemodynamic analysis was conducted on aneurysms for two stages (Table 2). As for morphological parameters, *H*_max_, *H*, *D*_middle_, *D*_neck_, volume, and surface area increased, while the diameter of parent artery decreased. Therefore, the AR and SR increased. Both *θ*_F_ and *θ*_A_ exceed the threshold value and UI, NSI, and EI increased in 2021. For hemodynamics, OSI and RRT increased, while NWSS was smaller. OSI was 0.0049 in 2019, which exceeded the threshold. RRSH and RRSC were greater than 30% in 2021, while RRSM was less than 30%. All three RRS values were less than 30% in 2019.

**Table 2:** Morphological and hemodynamics parameters of the aneurysm in two stages in case 2.

	Parameters	Time (year)		Threshold
		2019	2021	
Morphology parameters of aneurysms	*H*_max_ (mm)	2.968	4.905	/
	*H* (mm)	2.145	4.032	/
	*D*_middle_ (mm)	4.944	5.194	/
	*D*_neck_ (mm)	4.981	5.246	/
	*D*_vessel_ (mm)	4.295	3.727	/
	*V* (mm^3^)	27.768	48.090	/
	*S* (mm^2^)	35.333	58.129	/
	*θ*_F_ (deg)	/	153.45	>118.25
	*θ*_A_ (deg)	/	125.42	>96.1
	AR (H/*D*_neck_)	0.431	0.769	>1.6
	SR (*H*_max_/*D*_vessel_)	0.691	1.316	>1.75
	UI	0.018	0.131	>0.09
	EI	0.001	0.094	>0.13
	NSI	0.004	0.127	>0.16
Hemodynamic parameters of aneurysms	NWSS	0.958	0.487	<0.39
	OSI	0.0049	0.0157	>0.0036
	RRT	0.312	0.808	>2.7
Rupture Resemblance Scores	RRS_M_	6.73%	13.52%	>30%
	RRS_H_	7.60%	74.2%	>30%
	RRS_C_	3.99%	42.35%	>30%

## 4 Discussions

### 4.1 SR, AR, *θ*_F_, *θ*_A_, UI, EI, and NSI

At present, there have been many studies about the morphological changes of IAs after rupture (16, 24–27), and morphological parameters have also been enriched with the deepening of research. Based on previous research, the 3 IAs mentioned above were analyzed by AR, SR, *θ*_F_, *θ*_A_, UI, EI, NSI, and other morphological parameters.

Among the above analysis parameters, SR was previously considered most related to aneurysm rupture because it simultaneously focuses on the relationship between aneurysm size and the diameter of the parent artery. Kashiwazaki et al. (24) and Tremmel et al. (28) proposed that SR can highly predict the rupture state of small aneurysms (<5 mm). Our analysis shows that SR of the ruptured aneurysm was far greater than that of the unruptured aneurysm in Case 1. It even exceeds the threshold value of rupture risk. Besides, Dhar et al. (20) proposed that aneurysms are at risk of rupture when SR is greater than 2.05, by an analysis of SR of ruptured and unruptured aneurysms. This is similar to the analysis results of Case 1. The uniqueness of this case is in the fact that it involves a pair of mirrored aneurysms. The CT findings and CTA results alone are insufficient to fully determine the responsible aneurysm for SHA. Nevertheless, the assessment of the SR value can aid in identifying the responsible aneurysm and formulating an optimal surgical treatment plan.

However, although the SR of the aneurysm in case 2 increased by about one time during 2 years, it did not exceed the threshold value of rupture risk after the rupture. But it is worth noting that SR changes significantly. According to these analysis results, we are currently unsure of the accuracy and effectiveness of SR in predicting the risk of aneurysm rupture. Although no research currently focuses on the impact of the dynamic changes of SR on aneurysm rupture, we suggest that clinicians should be aware that an increasing trend of SR during follow-up may indicate that the aneurysm is developing toward rupture.

It seems inaccurate to assess the rupture risk of aneurysms only from SR of aneurysms. Some studies (29, 30) consider that AR is another parameter to predict the risk of aneurysm rupture. Weir et al. (29) shows that 88% of ruptured aneurysms had an AR > 1.6, while 56% of unruptured aneurysms had an AR ≤ 1.6. However, all IAs’ AR did not exceed the threshold value in our study. Although our research results differ from previous studies because we analyzed only three aneurysms in two cases, our results cannot deny the predictive value of AR for the risk of IA rupture. Besides, Yin et al. (31) believe that there is a U-shaped correlation between AR and the risk of aneurysm rupture, with a negative correlation range of 1.08 < AR ≤ 1.99 and a positive correlation range of 3.42 < AR ≤ 4.08. Further research with a larger sample size is needed to validate the ability of AI to assess the risk of aneurysm rupture. AR does only predict the rupture risk of IAs from the shape of the aneurysm itself, ignoring the impact of the parent artery on the aneurysm rupture. Further research with a larger sample size is needed to validate the ability of AR to assess the risk of aneurysm rupture.

The positional relationship between the parent artery and the aneurysm can often be reflected by *θ*_F_ and *θ*_A_. The growth direction and the thin-wall regions of an aneurysm can be inferred by *θ*_F_ and *θ*_A_, which affect the changes of aneurysms, and assess the risk of aneurysm rupture. At present, there are not many analytical studies on *θ*_F_ and *θ*_A_, and different studies on the correlation between aneurysm rupture and *θ*_F_ and *θ*_A_ have not maintained a consistent view (21, 32, 33). According to the analysis of this study, after the rupture of the aneurysm in case 2, *θ*_F_ and *θ*_A_ were greater than the threshold value of the rupture risk. While in case 1, the ruptured aneurysm’s *θ*_A_ was less than the threshold value of the rupture risk. Our results are different from those of Zheng et al. (33) and Dhar et al. (20). The research results of Bahagoglu et al. (21) inclined that *θ*_F_ is an important indicator to judge the rupture risk of an aneurysm, which is more consistent with our research results. *θ*_F_ and *θ*_A_, as morphological parameters, have been studied relatively few so far. Their effects on the growth pattern and the role on the rupture risk prediction of IAs remain controversial and have great potential research value.

As we all know, EI, UI, and NSI are commonly used to describe the shape characteristics of IAs. The previous studies (19, 34) have shown that ruptured aneurysms have characteristics of high UI, EI, and NSI. The analysis results of the 3 aneurysms in this paper were very consistent with previous studies. The UI, EI, and NSI of ruptured and unruptured aneurysms were significantly different. Particularly in case 2, the UI, EI, and NSI of the aneurysm were significantly increased, and the UI even exceeded the threshold value of the rupture risk. Therefore, it is reasonable to assume that UI, NSI, and EI have a high predictive value for the rupture risk of IAs.

### 4.2 WSS, OSI, and RRT

In the last 10 years, an increasing number of studies have recognized that hemodynamic changes in aneurysms are greatly related to the rupture of IAs, and studies based on CFD analysis of aneurysm hemodynamics are also very extensive (13, 19, 20, 35–44). WSS is one of the most studied hemodynamic parameters in recent years. But different studies hold different views on the specific role of WSS in predicting the rupture risk of IAs, so many scholars still doubt the effectiveness of WSS in predicting the rupture risk of IAs. There are two claims regarding the effect of WSS on aneurysm growth and rupture. (1) High WSS (35–37) is closely associated with aneurysm rupture because high WSS on the aneurysmal wall stimulates abnormal remodeling of endothelial cells, leading to aneurysm growth and ultimately aneurysm rupture. (2) Low WSS (13, 19, 37–41) is related with aneurysm rupture because low WSS on the aneurysm wall disrupts the aneurysmal rupture by provoking an inflammatory response that prompts endothelial cell degeneration. They speculated that high WSS and low WSS may, respectively, play different mechanisms in different stages of aneurysm initiation and progression. In order to make the comparison more referential, we use the NWSS value obtained by normalizing WSS via the parent artery. From the results of the aneurysm analysis of the two cases in this study, it can be seen that the ruptured aneurysm of case 1 had slightly higher NWSS than the unruptured aneurysm before rupture. Unfortunately, we did not have the image of this case after the rupture to conduct hemodynamic analysis. However, in case 2, NWSS was significantly lower after the aneurysm rupture. It is evident that the rupture of the aneurysms in these two cases seems to be influenced by high and low NWWS, respectively. in our opinion, limited by case screening and image acquisition, investigators focusing more on the appearance of reduced WSS after aneurysm rupture, but ignoring the dynamic changes of WSS throughout the growth of aneurysms.

Other hemodynamic parameters, such as OSI and RRT, have been proposed based on WSS studies, and have received attention because of their predictive value of aneurysm rupture risk. OSI in particular, demonstrated by numerous studies (25, 42–45), has been identified as helpful in predicting the risk of aneurysm rupture. Lu et al. (42) studied 9 pairs of mirrored aneurysms, and they suggested that the mean OSI in the ruptured group was 4 times that in the unruptured group. Their findings are very consistent with the results of aneurysm analysis in this study. Therefore, we believe that OSI may be effective and accurate in predicting the risk of aneurysm rupture. At present, the research on RRT is limited. Riccardello et al. proposed that ruptured aneurysms have prolonged RRT compared with unruptured aneurysms (41). Lu et al. also proposed that prolonged RRT is associated with intracranial vascular atherosclerosis and that structural remodeling of the vessel wall is one of the causes of ruptured aneurysms (14). The prolongation of RRT is mostly accompanied by low WSS and high OSI in aneurysms, which predicts a disturbed blood flow status and leads to a long residence of blood flow. Blood flow with such characteristic may trigger a series of inflammatory reactions responsible for a series of changes in endothelial cells. All of the above claims are currently at the hypothesis stage, and further research and exploration are still needed regarding the specific effect of RRT on the aneurysm.

### 4.3 RRS

A logistic regression model can be developed based on the differences of morphologic and hemodynamic parameters including SR, WSS, and OSI between ruptured and unruptured IAs (46). The model has also been used to predict the rupture probability of unruptured aneurysms, thus measuring their similarity to ruptured aneurysms. This probability of rupture is also known as the RRS, which proposed by Xiang et al. (23). The three scoring modalities, RRSM, RRSH, and RRSC, were established based on different parameter combination. All three prediction models had high sensitivity and specificity, especially the RRSH had the highest sensitivity and specificity. Our results also well printed the conclusion of the study by Xiang et al. (23). Therefore, we believe that the rupture of IAs depends more on hemodynamic changes, which may also be one of the reasons why many IAs are small in size but still rupture in clinic practice. Although the RRSM is less sensitive than the RRSH, it still exhibits good predictive value for aneurysm rupture, which may contribute to the utilization of SR rather than size as a key factor. This also exemplifies the potential value of SR for rupture risk assessment of IAs. Therefore, it is an important issue for neurosurgeons to consider whether the guiding criteria of rupture risk assessment and surgical intervention of unruptured IAs should be shifted from aneurysm size to more comprehensive morphological and hemodynamic analysis.

## 5 Limitations

There are also many shortcomings in our study. First, the number of cases in our study was small and not fully comprehensive. We were only able to verify whether there was consistent with previous findings based on the available results and to propose corresponding hypotheses. Second, this study only involved the morphological and hemodynamic analysis of posterior communicating artery aneurysms, and whether IAs in other locations also have corresponding characteristics cannot be determined. Finally, our hemodynamic analysis model adopts some assumptions and cannot fully reflect the real situation, which might be one reason for the discrepancy between our analysis results with those of other investigators.

## 6 Conclusion

Based on the morphological and hemodynamic analysis of IAs, SR, and OSI may have predictive values for the risk of IA rupture. It is possible that WSS changes before and after IA rupture, yet the influence of high or low WSS on growth and rupture of IA remains unclear. Moreover, RRS is promising to be used in the clinical assessment of the rupture risk of IAs and to guide the formulation of treatment plans.

## Data availability statement

The raw data supporting the conclusions of this article will be made available by the authors, without undue reservation.

## Ethics statement

The studies involving humans were approved by the Ethics Committee of the First Affiliated Hospital of Chongqing Medical University. The studies were conducted in accordance with the local legislation and institutional requirements. The participants provided their written informed consent to participate in this study.

## Author contributions

YZ: Investigation, Writing – original draft, Data curation, Project administration. RZ: Formal analysis, Methodology, Software, Writing – original draft. XS: Writing – review & editing. XL: Conceptualization, Investigation, Writing – original draft. JX: Formal analysis, Methodology, Writing – review & editing. ZG: Funding acquisition, Writing – review & editing. HS: Writing - review & editing.

## Figures and Tables

**Table:** 

*H*_max_	Maximum aneurysm height. Maximum distance from the neck center to the sac surface of the aneurysm.
*H*	The vertical height of the aneurysm. The maximum vertical distance from the neck plane to the top of the sac of the aneurysm.
*D*_middle_	Maximum cross-section diameter parallel to aneurysm neck plane.
*D*_neck_	Diameter of the aneurysm neck plane.
*D*_vessel_	The diameter of the vessel of the parent artery.
*V*	Aneurysm volume.
*V*_ch_	The volume of the convex hull of an aneurysm. The convex hull is the smallest convex body that completely surrounds the aneurysm.
*S*	The surface area of the aneurysm sac.
*S*_ch_	The surface area the convex hull. Aneurysm vessel angle (*θ*_F_), angle between the centerline of the inlet of the parent artery and the maximum height of the aneurysm (inflow direction side). Aneurysm inclination angle (*θ*_A_), the angle between the line of maximum height and the plane of the neck of the aneurysm (inflow direction side).
SR	Size ratio (*H*_max_/*D*_vessel_). The ratio of the maximum height of an aneurysm to the diameter of the parent artery.
AR	Aspect ratio (*H*/*D*_neck_). Ratio of maximum vertical height to aneurysm neck diameter.
UI	Undulation index. UI = 1 − *V*/*V*_ch_. The difference between the volume of the aneurysmal bulge and the volume of the aneurysm divided by the volume of the aneurysmal bulge quantifies the concavity of the aneurysmal surface.
EI	Ellipticity index. EI = 1 − (18π)1/3 *V*_ch_2/3 Sch. Quantifying the deviation of aneurysm convex hull shape from the perfect hemisphere.
NSI	Non-sphericity Index. NSI = 1 − (18π)1/3 *V*2/3 S. Quantifying the degree of deviation of aneurysm shape from the perfect hemisphere.
WSS	Wall shear stress. Friction generated by the flow of blood along the aneurysm wall. $WSS=\frac{1}{T}\overset{T}{\underset{0}{\int }}\left|wss_{i}\right|dt\text{,}$ (wss_i_ is the instantaneous shear stress vector and *T* is the duration of the cycle).
NWSS	Normalized wall shear stress.
OSI	Oscillatory shear index. Dimensionless measurements of the alignment between instantaneous vectors of wall shear stress and time-averaged vectors of wall shear stress were taken to quantify changes in the direction of wall shear stress during cardiac cycles. $OSI=\frac{1}{2}\left\{1-\frac{\left|\int_{0}^{T}wss_{i}dt\right|}{\left|\int_{0}^{T}\left|wss_{i}\right|dt\right|}\right\}\text{,}$ (wss_i_ is the instantaneous WSS vector and *T* is the duration of the cycle).
RRT	Relative residence time. Residence time of elements in the blood near the vessel wall. $RRT=\frac{1}{\left(1-2\times OSI\right)\times WSS}=\frac{1}{\frac{1}{T}\left|\int_{0}^{T}wss_{i}dt\right|}\text{.}$
RRSM	Rupture resemblance score based on morphological parameters of the aneurysm.
RRSH	Rupture resemblance score based on hemodynamic parameters of the aneurysm.
RRSC	Rupture resemblance score based on both morphological and hemodynamic parameters of the aneurysm.

## References

[1] Nieuwkamp DJ Setz LE Algra A Linn FH de Rooij NK Rinkel GJ. Changes in case fatality of aneurysmal subarachnoid haemorrhage over time, according to age, sex, and region: a meta-analysis. Lancet Neurol. (2009) 8:635–42. doi: 10.1016/s1474-4422(09)70126-7, PMID: 19501022

[2] Kim JH Suh SH Chung J Oh YJ Ahn SJ Lee KY. Prevalence and characteristics of unruptured cerebral aneurysms in ischemic stroke patients. J Stroke. (2016) 18:321–7. doi: 10.5853/jos.2016.00164, PMID: 27488981 PMC5066432

[3] Vlak MH Algra A Brandenburg R Rinkel GJ. Prevalence of unruptured intracranial aneurysms, with emphasis on sex, age, comorbidity, country, and time period: a systematic review and meta-analysis. Lancet Neurol. (2011) 10:626–36. doi: 10.1016/S1474-4422(11)70109-0 21641282

[4] Malhotra A Wu X Geng B Hersey D Gandhi D Sanelli P. Management of small unruptured intracranial aneurysms: a survey of neuroradiologists. AJNR Am J Neuroradiol. (2018) 39:875–80. doi: 10.3174/ajnr.A5631, PMID: 29650787 PMC7410652

[5] Suzuki T Takao H Rapaka S Fujimura S Ioan Nita C Uchiyama Y. Rupture risk of small unruptured intracranial aneurysms in Japanese adults. Stroke. (2020) 51:641–3. doi: 10.1161/strokeaha.119.027664, PMID: 31813355

[6] Chang HS. A theoretical consideration of the surgical treatment of small unruptured intracranial aneurysms. World Neurosurg. (2016) 96:302–8. doi: 10.1016/j.wneu.2016.09.017, PMID: 27641262

[7] Thompson BG Brown RD Amin-Hanjani S Broderick JP Cockroft KM Connolly ES. Guidelines for the management of patients with unruptured intracranial aneurysms: a guideline for healthcare professionals from the American Heart Association/American Stroke Association. Stroke. (2015) 46:2368–400. doi: 10.1161/str.0000000000000070 26089327

[8] Chmayssani M Rebeiz JG Rebeiz TJ Batjer HH Bendok BR. Relationship of growth to aneurysm rupture in asymptomatic aneurysms ≤ 7 mm: a systematic analysis of the literature. Neurosurgery. (2011) 68:1164–71. doi: 10.1227/NEU.0b013e31820edbd3, PMID: 21307791

[9] van Donkelaar CE Bakker NA Veeger NJ Uyttenboogaart M Metzemaekers JD Eshghi O. Prediction of outcome after subarachnoid hemorrhage: timing of clinical assessment. J Neurosurg. (2017) 126:52–9. doi: 10.3171/2016.1.Jns152136, PMID: 27035175

[10] Zheng J Xu R Guo Z Sun X. Small ruptured intracranial aneurysms: the risk of massive bleeding and rebleeding. Neurol Res. (2019) 41:312–8. doi: 10.1080/01616412.2018.1563737, PMID: 30638157

[11] Hassan T Timofeev EV Saito T Shimizu H Ezura M Matsumoto Y. A proposed parent vessel geometry-based categorization of saccular intracranial aneurysms: computational flow dynamics analysis of the risk factors for lesion rupture. J Neurosurg. (2005) 103:662–80. doi: 10.3171/jns.2005.103.4.0662, PMID: 16266049

[12] Sugiyama S Endo H Omodaka S Endo T Niizuma K Rashad S. Daughter sac formation related to blood inflow jet in an intracranial aneurysm. World Neurosurg. (2016) 96:396–402. doi: 10.1016/j.wneu.2016.09.040, PMID: 27647032

[13] Rashad S Sugiyama SI Niizuma K Sato K Endo H Omodaka S. Impact of bifurcation angle and inflow coefficient on the rupture risk of bifurcation type basilar artery tip aneurysms. J Neurosurg. (2018) 128:723–30. doi: 10.3171/2016.10.Jns161695, PMID: 28298037

[14] Sugiyama S Niizuma K Nakayama T Shimizu H Endo H Inoue T. Relative residence time prolongation in intracranial aneurysms: a possible association with atherosclerosis. Neurosurgery. (2013) 73:767–76. doi: 10.1227/neu.0000000000000096, PMID: 23863763

[15] Gholampour S Mehrjoo S. Effect of bifurcation in the hemodynamic changes and rupture risk of small intracranial aneurysm. Neurosurg Rev. (2021) 44:1703–12. doi: 10.1007/s10143-020-01367-3 32803404

[16] Tang X Zhou L Wen L Wu Q Leng X Xiang J. Morphological and hemodynamic characteristics associated with the rupture of multiple intracranial aneurysms. Front Neurol. (2021) 12:811281. doi: 10.3389/fneur.2021.811281, PMID: 35126301 PMC8812485

[17] Liang L Steinman DA Brina O Chnafa C Cancelliere NM Pereira VM. Towards the clinical utility of CFD for assessment of intracranial aneurysm rupture – a systematic review and novel parameter-ranking tool. J Neurointervent Surgery. (2019) 11:153–8. doi: 10.1136/neurintsurg-2018-014246, PMID: 30341160

[18] Saqr KM Rashad S Tupin S Niizuma K Hassan T Tominaga T. What does computational fluid dynamics tell us about intracranial aneurysms? A meta-analysis and critical review. J Cerebral Blood Flow Metab Off J Int Soc Cerebral Blood Flow Metabolism. (2020) 40:1021–39. doi: 10.1177/0271678x19854640, PMID: 31213162 PMC7181089

[19] Xiang J Natarajan SK Tremmel M Ma D Mocco J Hopkins LN. Hemodynamic-morphologic discriminants for intracranial aneurysm rupture. Stroke. (2011) 42:144–52. doi: 10.1161/strokeaha.110.592923, PMID: 21106956 PMC3021316

[20] Dhar S Tremmel M Mocco J Kim M Yamamoto J Siddiqui AH. Morphology parameters for intracranial aneurysm rupture risk assessment. Neurosurgery. (2008) 63:185–97. doi: 10.1227/01.Neu.0000316847.64140.81, PMID: 18797347 PMC2570753

[21] Baharoglu MI Schirmer CM Hoit DA Gao BL Malek AM. Aneurysm inflow-angle as a discriminant for rupture in sidewall cerebral aneurysms: morphometric and computational fluid dynamic analysis. Stroke. (2010) 41:1423–30. doi: 10.1161/strokeaha.109.570770, PMID: 20508183

[22] Lu Y Leng X Zou R Chen Q Li W Zhou X. Non-contrast enhanced silent MR angiography to evaluate hemodynamics and morphology of unruptured intracranial aneurysms a comparative computational fluid dynamics study. J Neurointerv Surg. (2022) 2022:18901. doi: 10.1136/neurintsurg-2022-018901 35882551

[23] Xiang J Yu J Choi H Dolan Fox JM Snyder KV Levy EI. Rupture resemblance score (RRS): toward risk stratification of unruptured intracranial aneurysms using hemodynamic-morphological discriminants. J Neurointerv Surgery. (2015) 7:490–5. doi: 10.1136/neurintsurg-2014-011218, PMID: 24811740 PMC6383516

[24] Kashiwazaki D Kuroda S. Size ratio can highly predict rupture risk in intracranial small (<5 mm) aneurysms. Stroke. (2013) 44:2169–73. doi: 10.1161/strokeaha.113.001138, PMID: 23743979

[25] Xiang J Yu J Snyder KV Levy EI Siddiqui AH Meng H. Hemodynamic-morphological discriminant models for intracranial aneurysm rupture remain stable with increasing sample size. J Neurointerv Surgery. (2016) 8:104–10. doi: 10.1136/neurintsurg-2014-011477, PMID: 25488922 PMC4791310

[26] Neyazi B Swiatek VM Skalej M Beuing O Stein KP Hattingen J. Rupture risk assessment for multiple intracranial aneurysms: why there is no need for dozens of clinical, morphological and hemodynamic parameters. Ther Adv Neurol Disord. (2020) 13:1756286420966159. doi: 10.1177/1756286420966159, PMID: 33403004 PMC7739206

[27] Cai W Hu C Gong J Lan Q. Anterior communicating artery aneurysm morphology and the risk of rupture. World Neurosurg. (2018) 109:119–26. doi: 10.1016/j.wneu.2017.09.118 28958928

[28] Tremmel M Dhar S Levy EI Mocco J Meng H. Influence of intracranial aneurysm-to-parent vessel size ratio on hemodynamics and implication for rupture: results from a virtual experimental study. Neurosurgery. (2009) 64, 69:11231. doi: 10.1227/01.Neu.0000341529.11231.69 PMC2775481 19349824

[29] Weir B Amidei C Kongable G Findlay JM Kassell NF Kelly J. The aspect ratio (dome/neck) of ruptured and unruptured aneurysms. J Neurosurg. (2003) 99:447–51. doi: 10.3171/jns.2003.99.3.0447, PMID: 12959428

[30] Nader-Sepahi A Casimiro M Sen J Kitchen ND. Is aspect ratio a reliable predictor of intracranial aneurysm rupture? Neurosurgery. (2004) 54:1343–8. doi: 10.1227/01.neu.0000124482.03676.8b, PMID: 15157290

[31] Yin JH Su SX Zhang X Bi YM Duan CZ Huang WM. U-shaped association of aspect ratio and single intracranial aneurysm rupture in chinese patients: a cross-sectional study. Front Neurol. (2021) 12:731129. doi: 10.3389/fneur.2021.731129, PMID: 34803880 PMC8598388

[32] Xu J Yu Y Wu X Wu Y Jiang C Wang S. Morphological and hemodynamic analysis of mirror posterior communicating artery aneurysms. PLoS One. (2013) 8:e55413. doi: 10.1371/journal.pone.0055413 23383184 PMC3561240

[33] Zheng Y Xu F Ren J Xu Q Liu Y Tian Y. Assessment of intracranial aneurysm rupture based on morphology parameters and anatomical locations. J Neurointerv Surgery. (2016) 8:1240–6. doi: 10.1136/neurintsurg-2015-012112 26863105

[34] Ludwig CG Lauric A Malek JA Mulligan R Malek AM. Performance of radiomics derived morphological features for prediction of aneurysm rupture status. J Neurointerv Surg. (2021) 13:755–61. doi: 10.1136/neurintsurg-2020-016808, PMID: 33158993

[35] Cebral JR Mut F Weir J Putman C. Quantitative characterization of the hemodynamic environment in ruptured and unruptured brain aneurysms. AJNR Am J Neuroradiol. (2011) 32:145–51. doi: 10.3174/ajnr.A2419, PMID: 21127144 PMC3086563

[36] Sugiyama S Meng H Funamoto K Inoue T Fujimura M Nakayama T. Hemodynamic analysis of growing intracranial aneurysms arising from a posterior inferior cerebellar artery. World Neurosurg. (2012) 78:462–8. doi: 10.1016/j.wneu.2011.09.023, PMID: 22120259

[37] Can A Du R. Association of hemodynamic factors with intracranial aneurysm formation and rupture: systematic review and meta-analysis. Neurosurgery. (2016) 78:510–20. doi: 10.1227/neu.0000000000001083 26516819

[38] Boussel L Rayz V McCulloch C Martin A Acevedo-Bolton G Lawton M. Aneurysm growth occurs at region of low wall shear stress: patient-specific correlation of hemodynamics and growth in a longitudinal study. Stroke. (2008) 39:2997–3002. doi: 10.1161/strokeaha.108.521617, PMID: 18688012 PMC2661849

[39] Miura Y Ishida F Umeda Y Tanemura H Suzuki H Matsushima S. Low wall shear stress is independently associated with the rupture status of middle cerebral artery aneurysms. Stroke. (2013) 44:519–21. doi: 10.1161/strokeaha.112.675306, PMID: 23223503

[40] Fukazawa K Ishida F Umeda Y Miura Y Shimosaka S Matsushima S. Using computational fluid dynamics analysis to characterize local hemodynamic features of middle cerebral artery aneurysm rupture points. World Neurosurg. (2015) 83:80–6. doi: 10.1016/j.wneu.2013.02.012 23403347

[41] Riccardello GJ Shastri DN Changa AR Thomas KG Roman M. Influence of relative residence time on Side-Wall aneurysm inception. Neurosurgery. (2018) 83:574–81. doi: 10.1093/neuros/nyx433, PMID: 28945849

[42] Lu G Huang L Zhang XL Wang SZ Hong Y Hu Z. Influence of hemodynamic factors on rupture of intracranial aneurysms: patient-specific 3D mirror aneurysms model computational fluid dynamics simulation. AJNR Am J Neuroradiol. (2011) 32:1255–61. doi: 10.3174/ajnr.A2461, PMID: 21757526 PMC7966033

[43] Jing L Fan J Wang Y Li H Wang S Yang X. Morphologic and hemodynamic analysis in the patients with multiple intracranial aneurysms: ruptured versus unruptured. PLoS One. (2015) 10:e0132494. doi: 10.1371/journal.pone.0132494, PMID: 26147995 PMC4492509

[44] Zhang Y Jing L Liu J Li C Fan J Wang S. Clinical, morphological, and hemodynamic independent characteristic factors for rupture of posterior communicating artery aneurysms. Journal of neurointerventional surgery. (2016) 8:808–12. doi: 10.1136/neurintsurg-2015-011865 26253110

[45] Brinjikji W Chung BJ Jimenez C Putman C Kallmes DF Cebral JR. Hemodynamic differences between unstable and stable unruptured aneurysms independent of size and location: a pilot study. J Neurointerv Surgery. (2017) 9:376–80. doi: 10.1136/neurintsurg-2016-012327 27048958

[46] Rajabzadeh-Oghaz H Waqas M Veeturi SS Vakharia K Tso MK Snyder KV. A data-driven model to identify high-risk aneurysms and guide management decisions: the rupture resemblance score. J Neurosurg. (2020) 135:9–16. doi: 10.3171/2020.5.Jns193264, PMID: 32886911 PMC10193488

